# Response of Watermelon to Drought Stress and Its Drought-Resistance Evaluation

**DOI:** 10.3390/plants14091289

**Published:** 2025-04-24

**Authors:** Kaili Ren, Taoxia Tang, Weiping Kong, Yongquan Su, Yuping Wang, Hong Cheng, Yonggang Yang, Xiaoqin Zhao

**Affiliations:** 1 Institute of Vegetables, Gansu Academy of Agricultural Sciences, Lanzhou 730070, China; renkaili66@126.com (K.R.); tangtaoxia@126.com (T.T.); wpk33@126.com (W.K.); gssyq@sohu.com (Y.S.); yyg_868@163.com (Y.Y.); gszhxqing@sohu.com (X.Z.); 2 College of Horticulture, Gansu Agricultural University, Lanzhou 730070, China; wangyp@gsau.edu.cn

**Keywords:** watermelon, drought stress, drought resistance, comprehensive evaluation

## Abstract

This study investigated the response of watermelon seedlings to drought stress by assessing the growth, physiological, and biochemical indices using a pot-based continuous drought method. Drought stress indices, phenotypic plasticity indices, and membership function values were calculated, followed by a correlation analysis, principal component analysis, and cluster analysis, to comprehensively evaluate the drought resistance of 13 watermelon genotypes. The results revealed that drought stress significantly reduced the fresh and dry weights, root length, root area, root volume, root tips, and forks of watermelon seedlings. Additionally, drought significantly reduced the relative water content of leaves and increased the levels of osmotic-adjustment substances (soluble sugars, soluble proteins, proline, and starch). Persistent drought also modulated the activities of antioxidant enzymes (SOD, POD, and CAT), leading to oxidative stress through the accumulation of H_2_O_2_. Membrane damage, indicated by a significant increase in the MDA content and relative conductivity, was observed, adversely affecting seedling growth. Phenotypic plasticity indices indicated that watermelon exhibits strong adaptability to drought. Cluster analysis categorized the 13 genotypes into four groups: highly drought-resistant (14X5), drought-resistant (LK13, JLR, HXF1, 14X4, 14X1, and 14X6), low drought-resistant (21F05, JH1, JR3, 14X7, and 16F02), and drought-sensitive (16C07). This study provides valuable genetic resources for breeding drought-resistant watermelon varieties.

## 1. Introduction

Drought is one of the most significant meteorological disasters worldwide, with its severity exacerbated by climate change. Rising global temperatures have increased evaporation rates, expanded arid regions, and intensified the agricultural constraints imposed by drought [1,2]. As a major abiotic stressor, drought severely impedes plant growth and development, often resulting in substantial yield losses or even plant death [3,4,5,6,7,8]. To mitigate these effects, plants have evolved adaptive mechanisms collectively termed “drought resistance”, which encompass morphological and biochemical responses, such as enhanced root systems, reduced transpiration, and improved water-use efficiency [4,9,10]. These strategies operate through three primary mechanisms: (i) *escape* (accelerating reproductive cycles before stress becomes lethal), (ii) *avoidance* (maintaining high internal water content to prevent tissue damage), and (iii) *tolerance* (sustaining growth under low water availability) [11].

Drought tolerance varies significantly among plant species. Watermelon (*Citrullus lanatus* (Thunb.) Matsum. et Nakai), originating from tropical Africa, has developed pronounced drought adaptability through long-term evolution. Its deep-rooted system enables efficient water extraction from soil, while moderate drought conditions can even enhance sugar accumulation. China is the largest producer of watermelon globally, accounting for 49.14% of the world’s production area and 60.95% of the total output in 2023 [12]. Given its economic importance and inherent drought tolerance, breeding and the promotion of new drought-resistant varieties are crucial for sustainable agriculture. The breeding of drought-resistant varieties is inseparable from drought-resistant germplasm resources.

Drought-resistance-evaluation methods significantly influence phenotypic assessments. Prior studies have employed diverse approaches:

- PEG-simulated drought at the bud stage (e.g., rice (*Oryza sativa* L.), maize (*Zea mays* L.), pea (*Pisum sativum*), foxtail millet (*Setaria italica* L.), soybean (*Glycine max*), white poplar (*Populus alba* L.), *Elymus nutans*) [13,14,15,16,17,18,19,20,21],
- PEG-simulated drought at the seedling stage (e.g., wheat (*Triticum aestivum* L.), barley (*Hordeum vulgare*), maize, sugar beet (*Beta vulgaris* L.), quinoa (*Chenopodium quinoa* Wild.), *Elymus nutans*) [20,22,23,24,25,26,27,28],
- Pot water control at the seedling stage (e.g., cassava (*Manihot esculenta* Crantz), watermelon, *Gleditsia sinensis*, herbaceous plants (*Limonium bicolor*, *Agropyron mongolicum*, *Agropyron desertorum*, *Astragalus adsurgens*, *Mellilotus of ficinalis*, *Trifolium repens*, *Medicago sativa*, *Glycyrrhiza uralensis*, *Artemisia ordosica*, *Suaeda glauca*, *Althaea rosea*, *Agriophyllum squarrosum*), wheat, *Helleborus orientalis*, chrysanthemum (*Chrysanthemum*)) [29,30,31,32,33,34,35],
- Field natural drought screening (e.g., maize, wheat, soybean, potato (*Solanum tuberosum* L.), cotton (*Gossypium*), chickpea (*Cicer arietinum* L.)) [36,37,38,39,40,41,42,43,44].

Among these, pot water control at the seedling stage offers greater reproducibility and operational feasibility for drought-resistance phenotyping.

In this study, we employed a pot water control method at the seedling stage to analyze the physiological and biochemical responses of 13 watermelon genotypes under drought stress. Drought stress indices and phenotypic plasticity indices were calculated, followed by a comprehensive evaluation via correlation analysis, principal component analysis (PCA), and cluster analysis. Our findings provide a foundation for selecting drought-resistant watermelon genotypes to support future breeding efforts.

## 2. Results

### 2.1. Drought Injury Index of 13 Watermelon Genotypes

Under drought stress, watermelon plants exhibited wilting, leaf rolling, and browning, with some plants dying. The drought injury index (DI) was calculated for each genotype, with values ranging from 1.54 (14X6) to 3.65 (JR3) (Table 1). Genotypes 14X6, 14X4, 14X5, and 14X1 showed the lowest DI values, indicating higher drought tolerance.

### 2.2. Effects of Drought Stress on Growth Parameters

**Figure 1:**
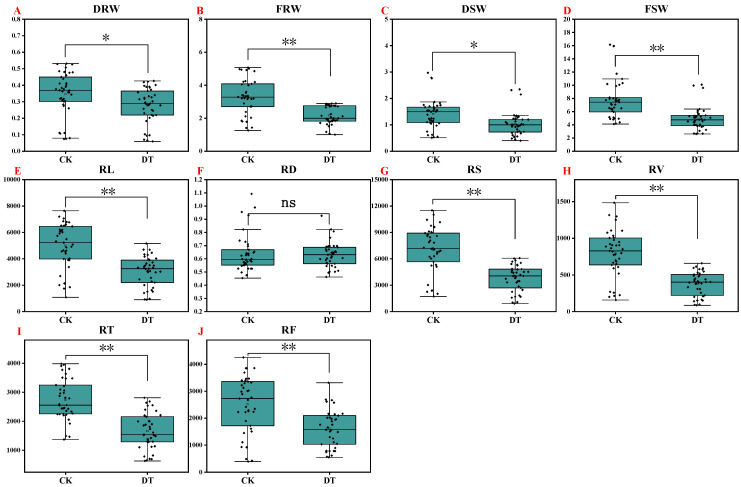
Box plots showing the descriptive statistics of the seedling growth traits. CK represents normal water control treatment, and DT represents drought stress treatment. Statistical significance was determined based on Tukey’s HSD, where ** *p* < 0.01, * *p* < 0.05, and ns means not significant. The horizontal line and square within the box represent the median and mean, respectively. The lower and upper limits of the box and lower and upper whiskers, represent Q1 (first quartile/25 percentile), Q3 (third quartile/75 percentile), (Q1 − 1.5 IQR), and (Q3 + 1.5 IQR), respectively. IQR-interquartile range. Black diamond dots on the boxes indicate the distribution of watermelon observations (DRW, root dry weight; FRW, root fresh weight; DSW, shoot dry weight; FSW, shoot fresh weight; RL, total root length; RD, average root diameter; RS, root surface area; RV, root volume; RT, root tips; RF, root forks). (**A**) Dry weight of root; (**B**) Fresh weight of root; (**C**) Dry weight of shoot; (**D**) Dry weight of shoot; (**E**) Total root length; (**F**) Average root diameter; (**G**) Root surface area; (**H**) Root volume; (**I**) Root tips; (**J**) Root forks.

**Figure 2:**
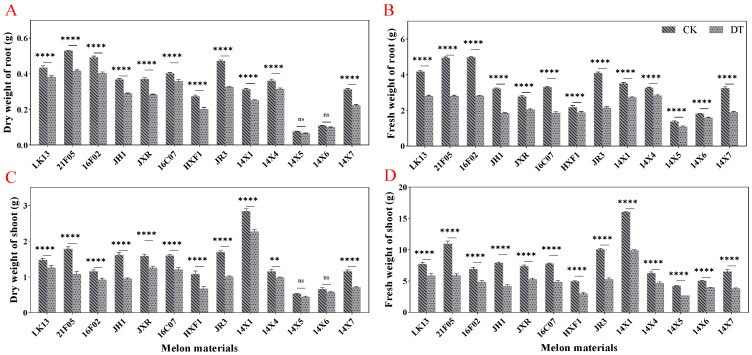
Effects of drought stress on fresh and dry weights of watermelon seedlings. (**A**) Dry weight of root. (**B**) Fresh weight of root. (**C**) Dry weight of shoot. (**D**) Fresh weight of shoot. CK represents normal water control treatment, and DT represents drought stress treatment. Statistically significant impacts and interactions, determined based on 2-way ANOVA, are indicated in each panel, where **** *p* < 0.0001, ** *p* < 0.01, and ns means not significant. Mean values ± standard errors are shown.

As we can see in Figure 1A–D, drought stress significantly reduced the growth of watermelon seedlings compared with the control. Due to drought treatment, a comparatively higher decrease in the seedling fresh weight was recorded than the seedling dry weight. The dry weights of the root and shoot were decreased by 19.99% and 27.33%, respectively, over the control, whereas 34.05% and 36.90% decreases were recorded in the fresh weights of the root and shoot, respectively, after drought treatment (Figure 1A–D). From a detailed view of different germplasms, drought stress significantly reduced the shoot and root biomass in most genotypes, except for 14X5 and 14X6, which maintained relatively stable dry weights of the shoot and root (Figure 2A–D).

**Figure 3:**
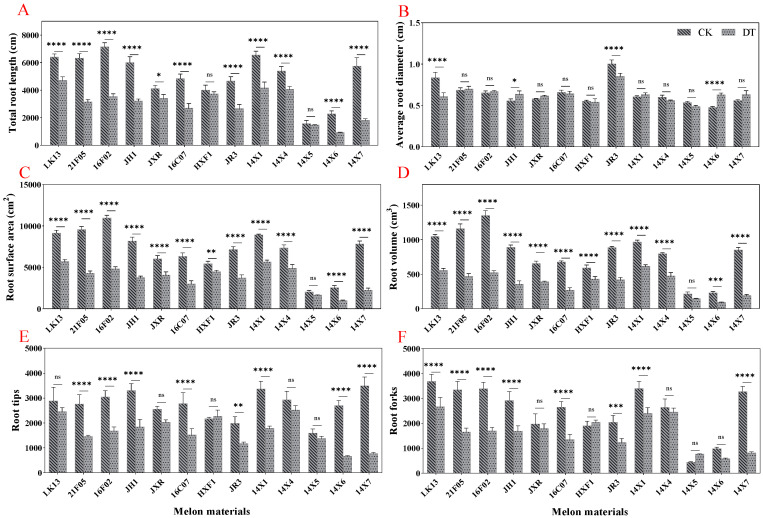
Effect of drought stress on root architecture of watermelon seedlings. (**A**) Total root length. (**B**) Average root diameter. (**C**) Root surface area. (**D**) Root volume. (**E**) Root tips. (**F**) Root forks. CK represents normal water control treatment, and DT represents drought stress treatment. Statistically significant impacts and interactions, determined based on 2-way ANOVA, are indicated in each panel, where **** *p* < 0.0001, *** *p* < 0.001, ** *p* < 0.01, * *p* < 0.05, and ns means not significant. Mean values ± standard error are shown.

Root architecture was also affected. In general, except for the root diameter, drought treatment significantly decreased the total root length, surface area, volume, tips, and forks of watermelon seedlings by 39.12%, 46.04%, 52.17%, 39.41%, and 35.28, respectively (Figure 1E–J). From the perspective of different germplasms, drought stress significantly reduced the total root length, surface area, and volume of most genotypes except for the total root length of HXF1 and the total root length, surface area, and volume of 14X5 (Figure 3A,C,D). The number of root tips and forks of watermelon seedlings also showed a downward trend after drought treatment, but the degree of the decline varied with different genotypes (Figure 3E,F). In addition, drought stress significantly increased the average root diameter of JH1 and 14X6 genotypes and significantly decreased the average root diameter of LK13 and JR3 genotypes (Figure 3B).

### 2.3. Effect of Drought Stress on Relative Water Content and Pigment Content

**Figure 4:**
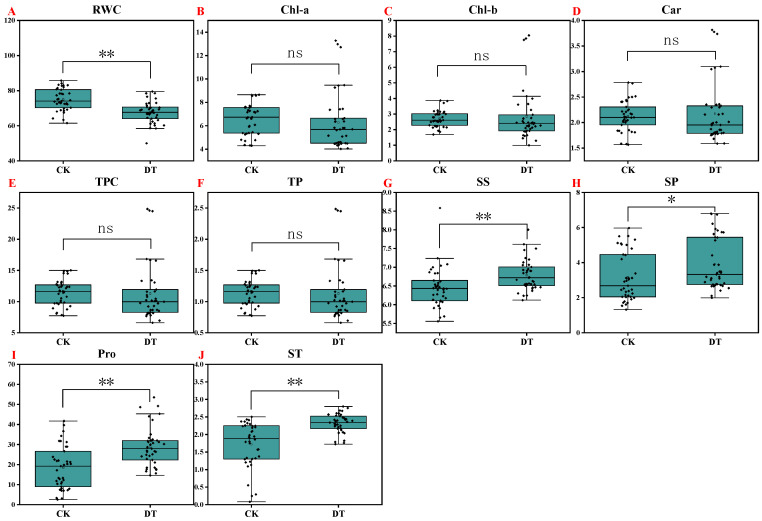
Box plots showing the descriptive statistics of some physiological and biochemical traits of watermelon seedlings. CK represents normal water control treatment, and DT represents drought stress treatment. Statistical significance was determined based on Tukey’s HSD, where ** *p* < 0.01, * *p* < 0.05, and ns means not significant. The horizontal line and square within the box represent the median and mean, respectively. The lower and upper limits of the box and lower and upper whiskers represent Q1 (first quartile/25 percentile), Q3 (third quartile/75 percentile), (Q1 − 1.5 IQR), and (Q3 + 1.5 IQR), respectively. IQR, interquartile range. Black diamond dots on the boxes indicate the distribution of watermelon observations (RWC, relative water content; Chl-a, chlorophyll a concentration; Chl-b, chlorophyll b concentration; Car, carotenoid concentration; TPC, total pigment concentration; TP, total pigment content; SS, soluble sugar content; SP, soluble protein content; Pro, proline content; St, starch content). (**A**) Relative water content; (**B**) Chl-a concentration; (**C**) Chl-b concentration; (**D**) Carotenoid concentration; (**E**) Total pigment concentration; (**F**) Total pigment content; (**G**) Soluble sugar content; (**H**) Soluble protein content; (**I**) Proline content; (**J**) Starch content.

**Figure 5:**
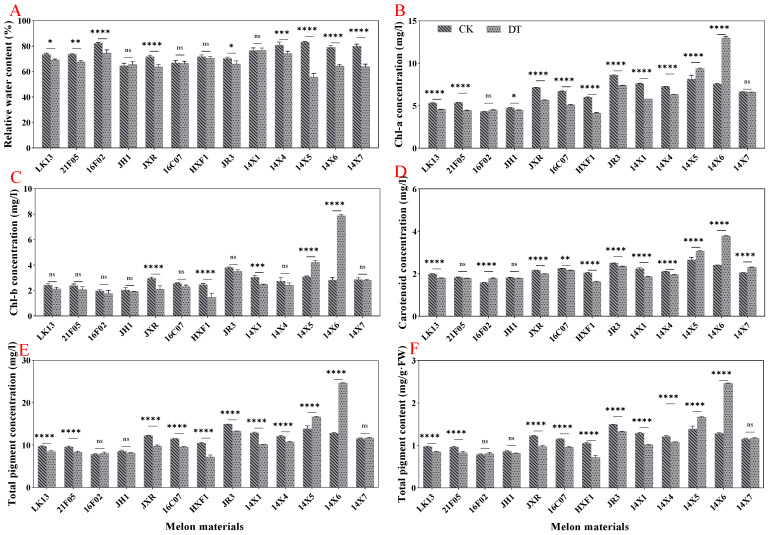
Effect of drought stress on relative water content and pigment content in watermelon leaves. (**A**) Relative water content. (**B**) Chl-a concentration. (**C**) Chl-b concentration. (**D**) Carotenoid concentration. (**E**) Total pigment concentration. (**F**) Total pigment content. CK represents normal water control treatment, and DT represents drought stress treatment. Statistically significant impacts and interactions, determined based on 2-way ANOVA, are indicated in each panel, where **** *p* < 0.0001, *** *p* < 0.001, ** *p* < 0.01, * *p* < 0.05, and ns means not significant. Mean values ± standard error are shown.

Compared with the control, drought stress significantly reduced the relative water content of leaves by 9.80% but had no significant effect on the pigment content (chlorophyll a concentration, chlorophyll b concentration, carotenoid concentration, total pigment concentration, and content) (Figure 4A–F). Judging from the germplasms of different genotypes, the relative water content in watermelon leaves decreased significantly under drought stress, except for JH1, 16C07, HXF1, and 14X1 genotypes (Figure 5A). The pigment content showed different degrees of increases and decreases under drought treatment. In general, under drought stress, the chlorophyll a concentration, chlorophyll b concentration, total pigment concentration, and content of 14X5 and 14X6 genotypes were significantly higher than those of the control, while the other genotypes showed different degrees of reductions or no significant difference (Figure 5B,C,E,F).

### 2.4. Effect of Drought Stress on the Contents of Osmotic Adjustment Substances

**Figure 6:**
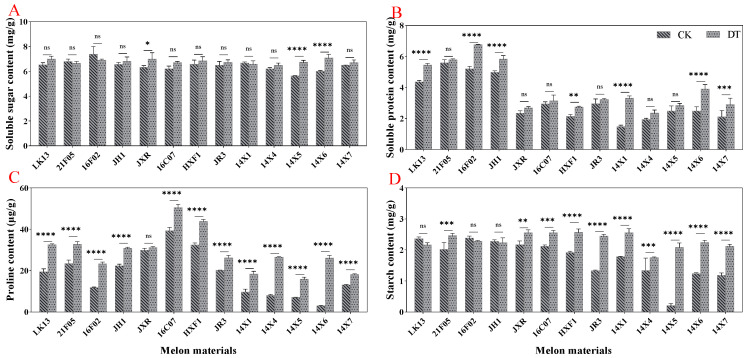
Effects of drought stress on osmotic-adjustment substances in watermelon leaves. (**A**) Soluble sugar content. (**B**) Soluble protein content. (**C**) Proline content. (**D**) Starch content. CK represents normal water control treatment, and DT represents drought stress treatment. Statistically significant impacts and interactions, determined based on 2-way ANOVA, are indicated in each panel, where **** *p* < 0.0001, *** *p* < 0.001, ** *p* < 0.01, * *p* < 0.05, and ns means not significant. Mean values ± standard error are shown.

When drought stress occurs in plants, the osmotic-adjustment substances tend to increase to reduce osmotic stress and improve the plant tolerance to drought stress. The results showed that compared with the control, drought stress significantly increased the content of soluble sugar, soluble protein, proline, and starch by 5.16%, 24.12%, 56.780%, and 34.46%, respectively (Figure 4G–J). From a detailed view of different germplasms, drought treatment significantly increased the soluble sugar content in JXR, 14X5, and 14X6 genotypes, significantly increased the soluble protein content in LK13, 16F02, JH1, HXF1, 14X1, 14X6, and 14X7 genotypes, and significantly increased the starch content in 21F05, JXR, 16C07, HXF1, JR3, 14X1, 14X4, 14X5, 14X6, and 14X7 genotypes (Figure 6A,B,D). It is worth noting that except for the JXR genotype, drought treatment significantly increased the proline content of the other watermelon genotypes (Figure 6C).

### 2.5. Antioxidant Enzyme Activities and Oxidative Stress

**Figure 7:**
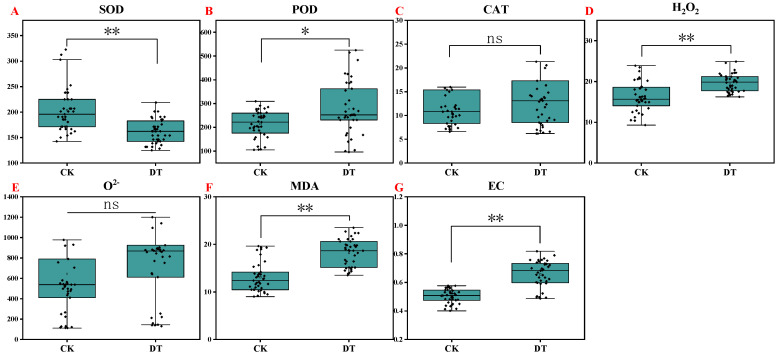
Box plots showing the descriptive statistics of some physiological and biochemical traits of watermelon seedlings. CK represents normal water control treatment, and DT represents drought stress treatment. Statistical significance was determined based on Tukey’s HSD, where ** *p* < 0.01, * *p* < 0.05, and ns means not significant. The horizontal line and square within the box represent the median and mean, respectively. The lower and upper limits of the box and lower and upper whiskers represent Q1 (first quartile/25 percentile), Q3 (third quartile/75 percentile), (Q1 − 1.5 IQR), and (Q3 + 1.5 IQR), respectively. IQR, interquartile range. Black diamond dots on the boxes indicate the distribution of watermelon observations (SOD, superoxide dismutase activity; POD, peroxidase activity; CAT, catalase activity; H_2_O_2_, hydrogen peroxide content; O^2−^, superoxide anion content; MDA, malonaldehyde content; REC, relative conductivity). (**A**) SOD activity; (**B**) POD activity; (**C**) CAT activity; (**D**) H_2_O_2_ content; (**E**) O^2−^ content; (**F**) MDA content; (**G**) Relative conductivity.

The activities of superoxide dismutase (SOD), peroxidase (POD), and catalase (CAT), and the contents of hydrogen peroxide (H_2_O_2_) and superoxide anion (O^2−^) in watermelon leaves were measured on the 7th day of continuous drought. The results showed that compared with the control, drought stress significantly reduced SOD activity (decreased by 20.52%) and increased POD activity and the H_2_O_2_ content (increased by 29.55% and 21.29%, respectively) but had no significant effect on CAT activity and the O^2−^ content (Figure 7A–E).

**Figure 8:**
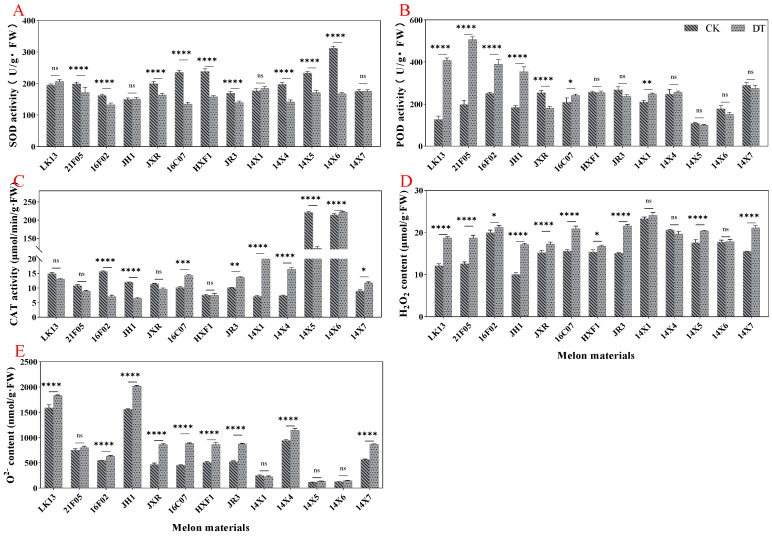
Effects of drought stress on antioxidant system in watermelon leaves. (**A**) SOD activity. (**B**) POD activity. (**C**) CAT activity. (**D**) H_2_O_2_ content. (**E**) O^2−^ content. CK represents normal water control treatment, and DT represents drought stress treatment. Statistically significant impacts and interactions, determined based on 2-way ANOVA, are indicated in each panel, where **** *p* < 0.0001, *** *p* < 0.001, ** *p* < 0.01, * *p* < 0.05, and ns means not significant. Mean values ± standard error are shown.

From the perspective of different germplasm resources, drought stress changed the activities of SOD, POD, and CAT, and the enzyme activities of some genotypes increased, while the enzyme activities of some genotypes decreased. Under drought stress, POD activity was significantly increased in the LK13, 21F05, 16F02, JH1, 16C07, and 14X1 genotypes but significantly decreased in the JXR genotype (Figure 8B). At the same time, CAT activity under drought stress was significantly increased in the 16C07, JR3, 14X1, 14X4, 14X6, and 14X7 genotypes and significantly decreased in the 16F02, JH1, and 14X5 genotypes (Figure 8C). However, drought stress significantly reduced the SOD activity of the 21F05, 16F02, JXR, 16C07, HXF1, JR3, 14X4, 14X5, and 14X6 genotypes (Figure 8A). What is more, drought stress significantly increased the H_2_O_2_ content in watermelon leaves except for the 14X1, 14X4, and 14X6 genotypes, and significantly increased the O^2−^ content in watermelon leaves, except for the 21F05, 14X1, 14X5, and 14X6 genotypes (Figure 8D,E). The increase in the active oxygen content indicated that drought stress caused oxidative stress.

### 2.6. Membrane Damage

**Figure 9:**
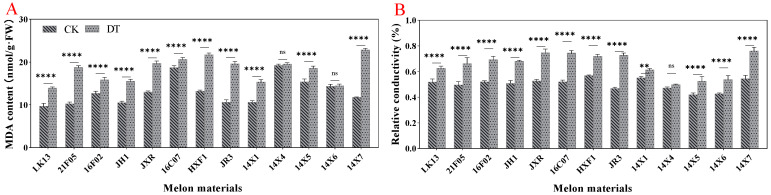
Effects of drought stress on membrane damage of watermelon leaves. (**A**) MDA content. (**B**) Relative conductivity. CK represents normal water control treatment, and DT represents drought stress treatment. Statistically significant impacts and interactions, determined based on 2-way ANOVA, are indicated in each panel, where **** *p* < 0.0001, ** *p* < 0.01, and ns means not significant. Mean values ± standard error are shown.

Malonaldehyde (MDA) and relative conductivity are important indexes to measure membrane damage. The results showed that drought stress significantly increased the MDA content and relative conductivity by 39.08%and 30.41%, respectively, compared with the control (Figure 7F,G). From the perspective of different germplasms, drought treatment increased the MDA content and relative conductivity of all watermelon genotypes (Figure 9A,B). Among them, the MDA content reached a significant level, except for the 14X4 and 14X6 germplasms, and the relative conductivity was significant except for the 14X4 germplasm.

### 2.7. Drought Stress Index and Phenotypic Plasticity Index

The drought stress index of 27 watermelon indexes under drought stress was calculated. The results are shown in Table 2. The drought stress indexes of 13 indexes (including the dry and fresh weights of the root, dry and fresh weights of the shoot, total root length, root surface area, root volume, root tips, root forks, relative water content, chlorophyll a concentration, total pigment concentration, and SOD activity) were less than 1, and 14 indexes (average root diameter, chlorophyll b concentration, carotenoid concentration, soluble sugar content, soluble protein content, proline content, starch content, MDA content, relative conductivity, POD activity, CAT activity, H_2_O_2_ content, O^2−^ content, and DI) were greater than 1. The variation in the drought stress index was abundant, and the coefficient of variation ranged from 6.979% to 121.634%. Among them, the coefficient of variation of the starch content was the largest, and the coefficient of variation of the soluble sugar content was the smallest.

In order to clarify the phenotypic plasticity of 27 traits of watermelon under drought stress, the phenotypic plasticity index was calculated, and the value was between 0.206 and 0.909 (Table 2). The phenotypic plasticity index of 15 traits (total root length, root surface area, root volume, root tips, root forks, chlorophyll a concentration, chlorophyll b concentration, total pigment concentration, soluble protein content, proline content, starch content, POD activity, CAT activity, O^2−^ content, and DI) were larger, ranging from 0.525 to 0.909, among which the phenotypic plasticity index of the starch content was the largest. From the phenotypic plasticity index of each index, watermelon has strong drought adaptability and can better adapt to environmental water changes.

### 2.8. Correlation Analysis

**Figure 10:**
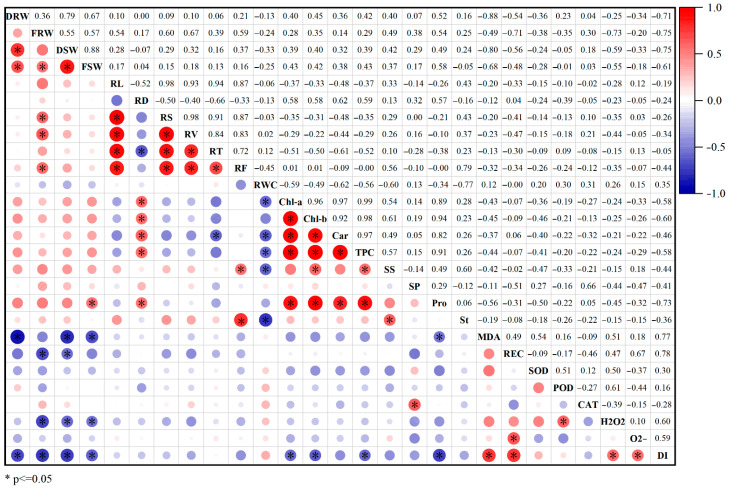
Correlation analyses of drought resistance index of watermelon under drought stress. In the lower panel, the red and blue circles indicate positive and negative correlations, respectively, with increasing size reflecting a higher coefficient. The upper panel shows the correlation coefficient of the related traits. * indicate significant at *p* ≤ 0.05 (DRW, root dry weight; FRW, root fresh weight; DSW, shoot dry weight; FSW, shoot fresh weight; RL, total root length; RD, average root diameter; RS, root surface area; RV, root volume; RT, root tips; RF, root forks; RWC, relative water content; Chl-a, chlorophyll a concentration; Chl-b, chlorophyll b concentration; Car, carotenoid concentration; TPC, total pigment concentration; SS, soluble sugar content; SP, soluble protein content; Pro, proline content; St, starch content; MDA, malonaldehyde content; REC, relative conductivity; SOD, superoxide dismutase activity; POD, peroxidase activity; CAT, catalase activity; H_2_O_2_, hydrogen peroxide content; O^2−^, superoxide anion content; DI, drought injury index).

The correlation analysis of the drought stress index of 27 traits of watermelon showed that there were different degrees of correlation between the drought stress indexes of each trait (Figure 10). The shoot fresh weight was significantly positively correlated with the root dry weight, root fresh weight, shoot dry weight, and proline content but significantly negatively correlated with the MDA content, H_2_O_2_ content, and DI. And the proline content was significantly positively correlated with the shoot fresh weight, average root diameter, chlorophyll a concentration, chlorophyll b concentration, carotenoid concentration, and total pigment concentration but significantly negatively correlated with MDA content and DI. In addition, the DI was significantly negatively correlated with dry and fresh weights of the root, dry and fresh weights of the shoot, chlorophyll a concentration, chlorophyll b concentration, total pigment concentration, and proline content (the correlation coefficients were −0.71, −0.75, −0.75, −0.61, −0.58, −0.60, −0.58, and −0.73, respectively) but significantly positively correlated with the MDA content, relative conductivity, H_2_O_2_, and O^2−^ content (the correlation coefficients were 0.77, 0.78, 0.60, and 0.59, respectively).

### 2.9. Principal Component Analysis

In order to reduce the redundancy of data, the drought stress indexes of 27 traits were analyzed via principal component analysis. According to the principle of an eigenvalue greater than 1, five principal components were extracted, and the original 27 single indexes were transformed into five new independent comprehensive indexes (PC1, PC2, PC3, PC4, and PC5). The eigenvalues of each principal component factor, the load matrix of the original index, and the contribution rate to the phenotype are shown in Table 3. The contribution rates of PC1, PC2, PC3, PC4, and PC5 factors were 33.900%, 25.108%, 13.067%, 8.681%, and 6.959%, respectively, and the cumulative contribution rate reached 87.715%. Among them, PC1 was mainly closely related to the plant biomass (dry and fresh weights of the root, dry and fresh weights of the shoot), relative water content, pigment concentration (chlorophyll a, chlorophyll b, carotenoid, and total pigment concentration), soluble sugar content, proline content, MDA content, relative conductivity, SOD activity, H_2_O_2_ content, and DI. PC2 was mainly related to the root architecture (total root length, average root diameter, root surface area, root volume, root tips, root forks) and pigment concentration (chlorophyll a, chlorophyll b, carotene, and total pigment concentration). PC3 was closely related to the relative water content, soluble sugar content, soluble protein content, starch content, relative conductivity, CAT activity, and O^2−^ content. PC4 was closely related to the POD activity and H_2_O_2_ content, and PC5 was closely related to the starch content, SOD activity, and O^2−^ content.

### 2.10. Comprehensive Evaluation of Drought Resistance

Based on the results of the principal component analysis, the membership function values of five comprehensive indexes were calculated (Table 4). The weights of the five principal component factors were calculated according to the contribution rate, and the weights of PC1 to PC5 were 0.3865, 0.2862, 0.1490, 0.0990, and 0.0793, respectively. According to the formula, the comprehensive evaluation value *D* of the drought resistance of each watermelon genotype was calculated. The drought resistance of 13 watermelon resources was ranked according to the *D* value, and the drought resistance from strong to weak was as follows: 14X5, 14X1, 14X6, 14X4, HXF1, LK13, JLR, 21F05, JH1, 14X7, JR3, 16F02, and 16C07 (Table 4).

**Figure 11:**
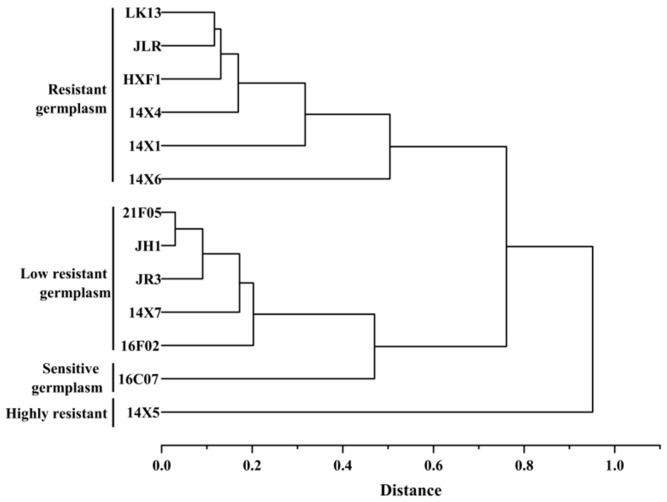
Systematic clustering of 13 watermelon genotypes based on *D*-values.

According to Ward’s method of squared deviations, the *D* value of watermelon was analyzed via cluster analysis. As shown in Figure 11, 13 watermelon genotypes were clustered into 4 categories. The first category was the highly-drought-resistant germplasm, including 14X5; the second category was the drought-resistant germplasms, including LK13, JLR, HXF1, 14X4, 14X1, and 14X6; the third category was the low-drought-resistant germplasms, including 21F05, JH1, JR3, 14X7, and 16F02; the fourth category was the drought-sensitive germplasm, including 16C07.

## 3. Discussion

Global climate change has brought about extreme drought, and the constraints of drought on agriculture have also intensified. Breeding and the promotion of new drought-resistant varieties are the most economical and effective measures. The breeding of drought-resistant varieties is inseparable from drought-resistant germplasms. Therefore, the identification methods of plant drought resistance (simple and easy-to-operate), the plant response to drought stress (the physiological and biochemical), and the comprehensive evaluation of plant drought resistance are very important for the screening and identification of drought-tolerant germplasms.

### 3.1. Identification Method of Plant Drought Resistance

The drought resistance of plants is affected by the identification methods. At present, the identification methods of plant drought resistance include PEG-simulated drought at the bud stage [13,14,15,16,17,18,19,20,21], PEG-simulated drought at the seedling stage [20,22,23,24,25,26,27,28], the pot water control method at the seedling stage [29,30,31,32,33,34,35], and the field natural identification method [36,37,38,39,40,41,42,43,44]. In this paper, the drought resistance of 13 different genotypes of watermelon was identified using the pot water control method at the seedling stage. However, different identification methods have their own advantages and disadvantages. The field identification method is closer to the field environment but is affected by uncontrollable rainfall and humidity. The study of Cai et al. (2020) [22] showed that there was no significant difference in the adverse effects of PEG-simulated drought stress and water control drought stress on barley growth. However, the decrease in the soil water content caused by drought is a slow process, while PEG treatment rapidly causes osmotic stress, so PEG cannot fully simulate drought stress conditions. The pot water control method at seedling are suitable for the laboratory and greenhouse, which can effectively and accurately control water. Therefore, it is more referential and operable to identify the drought tolerance of watermelon using the pot water control method at the seedling stage.

### 3.2. Response of Plants to Drought Stress

Drought stress inhibits plant growth, and plants respond to drought stress through a series of morphological, physiological, and biochemical adaptive evolution. The root is the sensor of plants, which senses osmotic stress under drought stress and plays an important role in the mechanism of plant drought resistance. Mahmood et al. (2022) [45] showed that the root length, root volume, and root number are key indicators of drought resistance in cotton. The study of Guo et al. (2024) [46] showed that the root architecture of drought-resistant cotton varieties showed a significant increase in the average length of all lateral roots and a significant decrease in the average lateral root emergence angle, while the drought-sensitive cotton varieties showed the opposite trend. The results of this study showed that drought significantly inhibited the growth of watermelon roots and significantly reduced the total root length, root volume, root area, root tips, and forks. Due to the pot water control method used in this paper, drought stress has seriously inhibited the root growth of all tested watermelon genotypes when scanning the roots on the 7th day of continuous drought.

The changes in the osmotic-adjustment substance contents and antioxidant enzyme activity are the key indicators of plant responses to drought stress [31,45]. Previous studies have shown that the contents of osmotic-adjustment substances (soluble protein and proline) in *Gleditsia sinensis* and oak (*Quercus*) were significantly increased under drought stress [31,47]. The results of this study showed that drought stress significantly increased the contents of soluble sugar, soluble protein, proline, and starch, which was consistent with previous studies.

The activity of antioxidant enzymes determines the level of ROS, and high levels of ROS lead to plant membrane damage. Liu et al. (2023) [31] and Xiong et al. (2022) [47] reported that drought stress significantly increased the activities of SOD, POD, and CAT in *Gleditsia sinensis* and oak, while Islam et al. (2020) [48] reported that the activities of SOD, POD, and CAT in sugar beet decreased significantly under drought stress. However, the results of this study showed that the antioxidant enzyme activities of different watermelon genotypes showed different levels of increase or decrease. The reason for this result is that on the one hand, different genotypes of watermelon have different resistances to drought stress, but on the other hand, this paper only sampled and measured on the 7th day of continuous drought, so the result is reasonable. Drought stress causes oxidative stress and membrane damage in plants. Wang et al. (2024) [49] showed that drought stress significantly increased the accumulation of ROS and MDA contents in tobacco (*Nicotiana tabacum* L.) seedlings. What is more, Liu et al. (2023) [31] and Xiong et al. (2022) [47] also reported that the MDA content in *Gleditsia sinensis* and oak increased with the aggravation of drought. This is consistent with the results of this study that drought stress significantly increased the H_2_O_2_ and MDA contents of all watermelon genotypes.

### 3.3. Comprehensive Evaluation of Plant Drought Resistance

Plant drought resistance is a comprehensive biological trait controlled by multiple genes. A single index cannot directly reflect the drought resistance of plants. Therefore, a comprehensive evaluation of plant drought resistance using multiple indicators is one of the effective methods to identify plant drought resistance. Badr et al. (2020) [13] identified the drought resistance of maize and used the frequency of the 5% optimal traits and 5% worst traits to screen high-resistance and high-sensitivity maize germplasms. Liu et al. (2023) [31] used the drought resistance index (drought stress index in this paper) as the main index, combined with growth, leaf morphology, and photosynthetic physiological indexes, to evaluate and identify the drought resistance of *Gleditsia sinensis*. Zhang et al. (2021) [50] calculated the membership function values of six indexes and evaluated the drought resistance of *Iris germanica*. Li et al. (2023) [51] transformed 13 physiological and biochemical indexes into four independent comprehensive indexes via PCA and evaluated the drought resistance of lettuce using three evaluation methods (*D* value, CDC, and WDC), and the results showed that there was no significant difference among the three evaluation methods. In this paper, the drought stress index was calculated, and the drought stress index was analyzed via correlation analysis and PCA. The 27 traits were transformed into five principal component factors, and the membership function value and comprehensive evaluation *D* value were calculated. And the *D* value was used to evaluate the drought resistance. Finally, the 27 traits of 13 watermelon resources were comprehensively evaluated for drought resistance, and one highly-drought-resistant germplasm and six drought-resistant germplasms were screened.

For the identification of watermelon drought resistance, He et al. (2023) [30] calculated the membership function value of the relative change rate of 13 traits of watermelon germplasms and comprehensively evaluated the drought resistance of watermelon. The physiological and biochemical traits of watermelon investigated in this paper are as many as 27, which are more comprehensive and representative.

In addition, this paper calculated the drought stress index, phenotypic plasticity index, and membership function value, carried out correlation analysis, PCA, and cluster analysis, and finally obtained the comprehensive evaluation *D* value [45]. The evaluation method can more truly and effectively reflect the drought resistance of plants.

## 4. Materials and Methods

### 4.1. Plant Materials

In this study, 13 watermelon genotypes with diverse genetic backgrounds preserved in the germplasm resource bank of the Vegetable Research Institute of Gansu Academy of Agricultural Sciences were used as materials. Detailed material information is shown in Table 5.

### 4.2. Material Planting and Drought Stress Treatment

The experiment was carried out in the greenhouse of Langou Village, Jiuhe Town, Lanzhou City. Watermelon seeds were soaked for germination on 23 March 2024 and sowed in pots (12.5 cm in diameter/11.2 cm in height) on 25 March 2024. The drought-stress treatment was started on 25 April 2024 (when the seedlings grew to the four-leaf stage). The experiment used natural drought treatment, that is, continuous drought treatment using a pot water control. During the seedling period, normal water management was carried out. When the seedlings grew to the four-leaf stage, the seedlings with uniform growth were selected for treatment. Sixty seedlings with the same growth were selected from each material and divided into control and treatment groups, with 30 seedlings in each group. The treatment group was not watered for 7 days, while control plants were maintained at 75% field capacity. The growth and physiological and biochemical parameters were measured at the end of the stress period.

### 4.3. Measuring Indicators and Methods

#### 4.3.1. Drought Injury Grade and Drought Injury Index

The classification of the drought injury grade was based on He et al. (2023) [30], and the five levels of S0–S4 in the literature were changed to S1–S5 in turn.

Drought injury index (DI) = (1 × S1 + 2 × S2 + 3 × S3 + 4 × S4 + 5 × S5)/total number of plants, (1)

where S1–S5 are the number of plants corresponding to drought levels 1 to 5. The greater the average drought injury index, the worse the drought resistance.

#### 4.3.2. Seedling Growth Index

Three seedlings were randomly selected from each treatment. The seedlings were divided into aboveground and underground parts from the cotyledon internodes, and the weights of the shoot and root were weighed using a one-thousandth electronic scale. The root fresh weight was weighed after washing the root and wiping the surface moisture. The dry weight of the material was deactivated at 105 °C for 30 min and baked at 80 °C for 24 h to a constant weight.

#### 4.3.3. Root Architecture

Three seedlings were randomly selected from each treatment, and the roots were washed with clean water, and then, the root architecture was analyzed using a plant root analyzer HM-GXONE.

#### 4.3.4. Physiologic and Biochemical Index

Relative water content: Three leaves of the same node were selected for each treatment, and the fresh weight (FW), saturated weight (TW; saturated leaf after full immersion in water), and dry weight (DW) were weighted. Relative water content (RWC) = (FW − DW)/(TW − DW) × 100%.

Relative conductivity: Three leaves of the same node were selected for each treatment, and 10 leaf discs were taken with a 0.8 cm puncher and placed in a test tube, and then, 5 mL of deionized water was added. Put it into the vacuum pump to extract air for 10 min, and measure the initial conductance S1 after shaking well. After boiling the water bath for 30 min, the conductivity S2 was measured after cooling to room temperature. The conductivity of deionized water was used as the blank S0. Relative conductivity (REC) = (S1 − S0)/(S2 − S0) × 100%.

Three seedlings were randomly selected from each treatment, and one fully expanded leaf below the growth point was picked up and frozen in liquid nitrogen for the detection of physiological and biochemical indexes. The chlorophyll content was determined using ethanol extraction method.

SOD, POD, CAT, MDA, H_2_O_2_, O^2−^, soluble protein, soluble sugar, proline, and starch were measured using the kits of Suzhou Grace Biotechnology Co., Ltd. (Suzhou, China), and the product numbers were G0102F, G0108F, G0106F, G0110F, G0112F, G0116F, G0417F, G0501F, G0111F96, and G0507F, respectively. All were operated according to the kit instructions.

### 4.4. Comprehensive Evaluation of Drought Resistance

The drought stress index, phenotypic plasticity index, membership function value, and comprehensive evaluation *D* value were calculated.

The drought stress index (DS) was the relative value of the treatment and control, and the formula was as follows:

(2) $$DS=\frac{X_{DS}}{X_{CK}}$$

where *X*_DS_ represents the value of the drought stress treatment, and *X*_CK_ represents the value of the normal water control.

The phenotypic plasticity index (PPI) is the difference between the maximum drought stress index and the minimum drought stress index divided by the maximum drought stress index, and the formula was as follows:

(3) $$PPI=\frac{{Max}_{DS}-{Min}_{DS}}{{Max}_{DS}}$$

A PCA was performed on the drought stress index of all traits, and then, its membership function value U(*X_j_*) was calculated:

(4) $$U\left(X_{j}\right)=\frac{X_{j}-X_{min}}{X_{max}-X_{min}},j=1,2,\ldots \ldots ,n$$

where *X_j_* is the *jth* principal component value of each variety, and *X_max_* and *X_min_* are maximum and minimum values of *jth* principal component value, respectively.

(5) $$W_{j}=\frac{P_{j}}{\sum_{j=1}^{n}P_{j}},j=1,2,\ldots \ldots ,n$$

where *W_j_* represents the importance of the *jth* principal component, that is, the weight; *P_j_* represents the contribution rate of the *jth* principal component.

The *D* value is the comprehensive evaluation value. The higher the *D* is, the material is indicated as having greater comprehensive drought resistance. The calculation formula for the *D* value is as follows:

(6) $$D=\sum_{j=1}^{n}\left[U(X_{j})\times W_{j}\right],j=1,2,\ldots \ldots ,n$$

### 4.5. Statistics

Microsoft Excel was used for data collation, and SPSS 26 was used for significance analysis, PCA, etc. Prism 9 software was used to make the histogram, and Origin 2024 software was used to analyze the related heat map and cluster map.

## 5. Conclusions

(1) This paper clarified the growth and physiological and biochemical responses of watermelon seedlings under drought stress. Compared to the control, drought stress significantly reduced the fresh and dry weights, root length, root area, root volume, root tips, and forks of watermelon seedlings. What is more, drought significantly reduced the relative water content of leaves and increased the levels of osmotic-adjustment substances (soluble sugars, soluble proteins, proline, and starch). Persistent drought also modulated the activities of antioxidant enzymes (SOD, POD, and CAT), leading to oxidative stress through the accumulation of H_2_O_2_, resulting in oxidative stress. Membrane damage, indicated by a significant increase in the MDA content and relative conductivity, was observed, adversely affecting seedling growth.
(2) The 13 watermelon genotypes were clustered into 4 categories. The first category was a highly-drought-resistant germplasm, including 14X5; the second category was drought-resistant germplasms, including LK13, JLR, HXF1, 14X4, 14X1, and 14X6; the third category was low-drought-resistant germplasms, including 21F05, JH1, JR3, 14X7, and 16F02; the fourth category was a drought-sensitive germplasm, including 16C07. This study provides the material basis for watermelon drought resistance breeding.

## Figures and Tables

**Table 1:** Drought injury index of watermelon germplasm resources.

Germplasm	DI	Germplasm	DI
LK13	2.83	JR3	3.65
21F05	3.18	14X1	2.21
16F02	2.86	14X4	1.68
JH1	2.95	14X5	1.95
JLR	3.19	14X6	1.54
16C07	3.20	14X7	3.45
HXF1	3.14		

**Table 2:** Drought stress index and phenotypic plasticity index (PPI).

Traits	Drought Stress Index					PPI
	Min	Max	AVE	SE	CV	
DRW	0.712	0.897	0.807	0.069	8.503	0.206
FRW	0.521	0.868	0.688	0.135	19.603	0.400
DSW	0.589	0.867	0.736	0.113	15.333	0.320
FSW	0.523	0.786	0.643	0.090	14.030	0.334
RL	0.315	0.943	0.632	0.195	30.880	0.666
RD	0.729	1.323	1.009	0.146	14.471	0.449
RS	0.288	0.819	0.559	0.161	28.783	0.648
RV	0.224	0.726	0.497	0.145	29.240	0.691
RT	0.221	1.045	0.630	0.243	38.586	0.788
RF	0.250	1.752	0.739	0.374	50.613	0.857
RWC	0.670	1.015	0.907	0.097	10.693	0.340
Chl-a	0.691	1.712	0.944	0.263	27.854	0.597
Chl-b	0.598	2.786	1.041	0.552	53.053	0.786
Car	0.793	1.566	1.016	0.198	19.481	0.493
TPC	0.689	1.921	0.979	0.311	31.729	0.641
SS	0.934	1.196	1.056	0.074	6.979	0.219
SP	1.035	2.224	1.297	0.313	24.105	0.534
Pro	1.043	8.649	2.219	2.017	90.911	0.879
St	0.916	10.033	2.001	2.434	121.634	0.909
MDA	1.011	1.943	1.440	0.315	21.853	0.479
REC	1.055	1.553	1.304	0.135	10.329	0.321
SOD	0.538	1.057	0.821	0.174	21.177	0.491
POD	0.710	3.204	1.379	0.752	54.520	0.778
CAT	0.448	2.876	1.176	0.692	58.828	0.844
H2O2	0.954	1.708	1.256	0.238	18.976	0.441
O^2−^	0.929	1.956	1.378	0.324	23.548	0.525
DI	1.542	3.650	2.757	0.685	24.838	0.578

Note: Min, Max, AVE, SE, and CV represent the minimum, maximum, mean, standard error, and coefficient of variation of the drought stress index (DRW, root dry weight; FRW, root fresh weight; DSW, shoot dry weight; FSW, shoot fresh weight; RL, total root length; RD, average root diameter; RS, root surface area; RV, root volume; RT, root tips; RF, root forks; RWC, relative water content; Chl-a, chlorophyll a concentration; Chl-b, chlorophyll b concentration; Car, carotenoid concentration; TPC, total pigment concentration; SS, soluble sugar content; SP, soluble protein content; Pro, proline content; St, starch content; MDA, malonaldehyde content; REC, relative conductivity; SOD, superoxide dismutase activity; POD, peroxidase activity; CAT, catalase activity; H_2_O_2_, hydrogen peroxide content; O^2−^, superoxide anion content; DI, drought injury index).

**Table 3:** Component matrix and the cumulative contribution rate of principal components.

Evaluation Traits	PC1		PC2		PC3		PC4		PC5	
	Load Capacity	Weight	Load Capacity	Weight	Load Capacity	Weight	Load Capacity	Weight	Load Capacity	Weight
DRW	0.686	0.075	0.092	0.014	−0.217	−0.062	0.446	0.19	−0.394	−0.21
FRW	0.72	0.079	0.489	0.072	−0.147	−0.042	−0.276	−0.118	0.064	0.034
DSW	0.767	0.084	0.271	0.04	−0.24	−0.068	0.214	0.091	−0.219	−0.117
FSW	0.708	0.077	0.128	0.019	−0.226	−0.064	0.169	0.072	−0.401	−0.214
RL	0.115	0.013	0.952	0.14	0.235	0.067	0.015	0.007	−0.005	−0.003
RD	0.368	0.04	−0.626	−0.092	−0.055	−0.016	−0.416	−0.178	0.086	0.046
RS	0.151	0.016	0.961	0.142	0.134	0.038	−0.052	−0.022	0.074	0.039
RV	0.219	0.024	0.922	0.136	0.014	0.004	−0.138	−0.059	0.111	0.059
RT	−0.073	−0.008	0.934	0.138	0.157	0.044	0.135	0.058	−0.153	−0.081
RF	0.409	0.045	0.768	0.113	0.4	0.113	0.066	0.028	0.251	0.134
RWC	−0.549	−0.06	0.088	0.013	−0.529	−0.15	−0.12	−0.051	−0.427	−0.227
Chla	0.788	0.086	−0.541	−0.08	0.168	0.048	0.096	0.041	0.124	0.066
Chlb	0.811	0.089	−0.501	−0.074	0.13	0.037	0.013	0.005	0.049	0.026
Car	0.71	0.078	−0.647	−0.095	0.239	0.068	0.065	0.028	0.063	0.033
TPC	0.797	0.087	−0.545	−0.08	0.164	0.047	0.063	0.027	0.089	0.047
SS	0.662	0.072	0.111	0.016	0.553	0.157	0.027	0.011	0.001	0
SP	0.286	0.031	−0.018	−0.003	−0.705	−0.2	−0.336	−0.143	0.391	0.208
Pro	0.862	0.094	−0.376	−0.055	−0.095	−0.027	−0.074	−0.031	−0.068	−0.036
St	0.411	0.045	0.315	0.046	0.559	0.158	0.158	0.067	0.509	0.271
MDA	−0.763	−0.083	−0.169	−0.025	0.139	0.039	−0.119	−0.051	0.443	0.236
REC	−0.5	−0.055	−0.441	−0.065	0.632	0.179	−0.162	−0.069	−0.16	−0.085
SOD	−0.524	−0.057	−0.025	−0.004	−0.409	−0.116	0.344	0.147	0.505	0.269
POD	−0.309	−0.034	−0.035	−0.005	−0.304	−0.086	0.847	0.361	−0.048	−0.026
CAT	0.048	0.005	0.201	0.03	−0.673	−0.191	−0.472	−0.201	0.096	0.051
H_2_O_2_	−0.631	−0.069	−0.298	−0.044	0.201	0.057	0.526	0.224	0.055	0.029
O^2−^	−0.349	−0.038	0.036	0.005	0.549	0.156	−0.468	−0.2	−0.514	−0.274
DI	−0.898	−0.098	−0.173	−0.026	0.289	0.082	−0.08	−0.034	−0.116	−0.062
Eigen values	9.153		6.779		3.528		2.344		1.879	
Variance contribution (%)	33.900		25.108		13.067		8.681		6.959	
Cumulative variance contribution (%)	87.715									

Note: DRW, root dry weight; FRW, root fresh weight; DSW, shoot dry weight; FSW, shoot fresh weight; RL, total root length; RD, average root diameter; RS, root surface area; RV, root volume; RT, root tips; RF, root forks; RWC, relative water content; Chl-a, chlorophyll a concentration; Chl-b, chlorophyll b concentration; Car, carotenoid concentration; TPC, total pigment concentration; SS, soluble sugar content; SP, soluble protein content; Pro, proline content; St, starch content; MDA, malonaldehyde content; REC, relative conductivity; SOD, superoxide dismutase activity; POD, peroxidase activity; CAT, catalase activity; H_2_O_2_, hydrogen peroxide content; O^2−^, superoxide anion content; DI, drought injury index.

**Table 4:** Membership function values of five main factors and *D*-value ranks of drought tolerance.

Germplasm	U(X_1_)	U(X_2_)	U(X_3_)	U(X_4_)	U(X_5_)	*D*-Value	Tolerance Rank
LK13	0.26	0.74	0.40	1.00	−0.10	0.46	6
21F05	0.06	0.35	0.54	0.61	1.22	0.36	8
16F02	0.31	0.35	0.45	0.48	−0.16	0.32	12
JH1	0.10	0.33	0.56	0.55	0.97	0.35	9
JLR	0.26	0.75	0.75	0.12	−0.38	0.41	7
16C07	0.22	0.46	0.66	0.26	−2.29	0.16	13
HXF1	0.19	1.00	0.66	0.00	0.29	0.48	5
JR3	0.00	0.43	0.78	0.15	0.92	0.33	11
14X1	0.32	0.74	0.00	0.08	2.43	0.54	2
14X4	0.53	0.88	0.31	0.29	−0.44	0.50	4
14X5	0.67	0.94	1.00	0.57	2.72	0.95	1
14X6	1.00	0.00	0.56	0.23	0.14	0.50	3
14X7	0.11	0.05	0.61	0.16	2.11	0.33	10

Note: U(X_1_) to U(X_5_) represent the membership function values of the first principal component to the fifth principal component, respectively. The *D*-value is the comprehensive evaluation value.

**Table 5:** Thirteen watermelon germplasms with different genotypes.

Name	Fruit Shape	Peel Covering Type	Flesh Color	Seed Morphology	Type	Abbreviation
Long Ke 13	Ellipse	Green peel	Red	Brown small seed	Big fruit size	LK13
2021F05	Roundness	Green peel	Pink	dark brown middle seed	Small fruit type	21F05
2016F02	Roundness	Green peel	Red	dark brown middle seed	Small fruit type	16F02
Jin Hua 1	Ellipse	Green peel	Red	Brown small seed	Big fruit size	JH1
Jiao Li Ren	Roundness	Green peel	Yellow	Black small seed	Small fruit type	JLR
2016C07	Roundness	Green peel	Red	Black small seed	Small fruit type	16C07
Hua Xin F1	Roundness	Green peel	Pink	Black small seed	Small fruit type	HXF1
JR3	Roundness	Black peel	Yellow	Black middle seed	Seed watermelon	JR3
2014X1	Roundness	Green peel	Faint yellow	Red middle seed	Wild watermelon	14X1
2014X4	Roundness	Green peel	Faint yellow	Yellow medium seed	Wild watermelon	14X4
2014X5	Roundness	Green peel	White	White medium seed	Wild watermelon	14X5
2014X6	Roundness	Green peel	White	Yellow medium seed	Wild watermelon	14X6
2014X7	Roundness	Walnut peel	White	Yellow big seed	Wild watermelon	14X7

## Data Availability

All relevant data are within the paper.

## References

[1] Zhu Y.L., Liu Y., Kong X.H., Wang X.X., Zhang M.Q., Hong X.W., Chen H.P., Sun J.Q. (2025). Research progress and prospect on the drought, heatwave, and compound drought and heatwave events in China. Trans. Atmos. Sci..

[2] Chen Y.N., Li Z., Fang G.H., Li Y.P. (2025). Global drought variation and its adaptation. Sci. Technol. Rev..

[3] Cao Y., Yang W., Ma J., Cheng Z., Zhang X., Liu X., Wu X., Zhang J. (2024). An Integrated framework for drought stress in plants. Int. J. Mol. Sci..

[4] Hura T., Hura K., Ostrowska A. (2022). Drought-stress induced physiological and molecular changes in plants. Int. J. Mol. Sci..

[5] Li Y.Y., Zhao X., Yue L., Qiu Y. (2024). Research progress and prospect on the interaction between plant root exudates and rhizosphere microorganisms under drought stress. J. Arid Meteorol..

[6] Mukherjee A., Dwivedi S., Bhagavatula L., Datta S. (2023). Integration of light and ABA signaling pathways to combat drought stress in plants. Plant Cell Rep..

[7] Zhang C.Q., Li L.L. (2024). Citespace-based analysis of the effects of high temperature and drought on plant growth and metabolism. J. Mt. Agric. Biol..

[8] Zhang H., Zhu J., Gong Z., Zhu J.K. (2022). Abiotic stress responses in plants. Nat. Rev. Genet..

[9] Gupta A., Rico-Medina A., Caño-Delgado A.I. (2020). The physiology of plant responses to drought. Science.

[10] Hura T., Dziurka M., Hura K., Ostrowska A., Dziurka K. (2016). Different allocation of carbohydrates and phenolics in dehydrated leaves of triticale. J. Plant Physiol..

[11] Basu S., Ramegowda V., Kumar A., Pereira A. (2016). Plant adaptation to drought stress. F1000Research.

[12] FAOSTAT Data/QCL. https://www.fao.org/faostat/en/.

[13] Badr A., El-Shazly H.H., Tarawneh R.A., Börner A. (2020). Screening for drought tolerance in maize (*Zea mays* L.) germplasm using germination and seedling traits under simulated drought conditions. Plants.

[14] Hou S., Qin H.B., Li M., Wang H.G., Mu Z.X. (2024). Identification of agronomic traits and drought tolerance at germination stage of 44 local foxtail millet varieties from Shanxi Province. Agric. Res. Arid Areas.

[15] Huang Y.X., Liu X., Yan S.J., Li N., Zhang X.W., Liu Z., Wang W.P. (2024). Evaluation and screening of rice germplasm resources for drought resistance in germination stage. Hybrid Rice.

[16] Li L., Shen B.Y., Zhang T.J., Yang T., Liu R., Zong X.X. (2017). Evalation and screening of pea (*Pisum Sativum*) germplasm resources for drought resistance during germination stage. J. Plant Genet. Resour..

[17] Xie J.G., Wang M.L., Zhang Y.F., Meng F.F., Zheng Y.H., Li G., Sun X.M., Fan X.H., Yang Z.Y., Wang S.M. (2025). QTL mapping and related gene mining of drought tolerance at germination stage in soybean. Chin. J. Oil Crop Sci..

[18] Zhao M., Yang S., Jiang H.W., Xie J.G., Zhou R., Zheng L.P., Meng F.F., Wang S.M. (2023). Identification and comprehensive evaluation of drought-tolerance soybean germplasm resources at bud stage. Soybean Sci..

[19] Zhao X., Liu Z., Li H., Zhang Y., Yu L., Qi X., Gao H., Li Y., Qiu L. (2022). Identification of Drought-tolerance genes in the germination stage of soybean. Biology.

[20] Long J., Dong M., Wang C., Miao Y. (2023). Effects of drought and salt stress on seed germination and seedling growth of *Elymus nutans*. PeerJ.

[21] Vuksanović V., Kovačević B., Kebert M., Pavlović L., Kesić L., Čukanović J., Orlović S. (2023). In vitro selection of drought-tolerant white poplar clones based on antioxidant activities and osmoprotectant content. Front. Plant Sci..

[22] Cai K., Chen X., Han Z., Wu X., Zhang S., Li Q., Nazir M.M., Zhang G., Zeng F. (2020). Screening of worldwide barley collection for drought tolerance: The assessment of various physiological measures as the selection criteria. Front. Plant Sci..

[23] Hou L.Y., Chen Y.H., Wang Y.C., Hong D.X., Wang Y.X., Chen Z., Wu S.J., Dong Y.H. (2024). Influence of drought stress duration on quinoa physiological characteristics and drought tolerance evaluation at seedling stage. J. Nucl. Agric. Sci..

[24] Wang L., Deng J., Zhang Z., Zhao M.W., Che X.Y., Wang G.Y., Guo X., Zhang H.Y., He L., Weng J.F. (2024). Identification and evaluation of drought tolerant germplasm resources at seedling stage of maize under PEG stress. Crops.

[25] Yang D.D., Han X., Kong X.X., Zhao P.F., Jin J.M., Zhao G.X., Su Y.Z., Zhao G.J. (2024). Identification and screening of drought tolerance in 76 winter wheat varieties (lines) during seedling stage. China Seed Ind..

[26] Mohi-Ud-Din M., Hossain M.A., Rohman M.M., Uddin M.N., Haque M.S., Ahmed J.U., Hossain A., Hassan M.M., Mostofa M.G. (2021). Multivariate analysis of morpho-physiological traits reveals differential drought tolerance potential of bread wheat genotypes at the seedling stage. Plants.

[27] Rida S., Maafi O., López-Malvar A., Revilla P., Riache M., Djemel A. (2021). Genetics of germination and seedling traits under drought stress in a MAGIC population of maize. Plants.

[28] Tan W., Li W., Li J., Liu D., Xing W. (2023). Drought resistance evaluation of sugar beet germplasms by response of phenotypic indicators. Plant Signal Behav..

[29] Chen M., Chen L., Li S.J., Xie H.H., Song Y. (2024). Effects of drought stress on physiological characteristics and stomatal morphology of different cassava seedlings. J. Sichuan Agric. Univ..

[30] He Y.P., Yin L.J., Ding X.L., Wang C.X., Hou Y.J., Ma Y.H., Yu R., Yue Z., Yang J.Q., Zhang X. (2023). Identification and screening of drought resistance indexes of 25 watermelon germplasms at seedling stage. J. Northwest A F Univ. Nat. Sci. Ed..

[31] Liu F., Zhao Y., Wang X., Wang B., Xiao F., He K. (2023). Physiological response and drought resistance evaluation of *Gleditsia sinensis* seedlings under drought-rehydration state. Sci. Rep..

[32] Xu A.Y., Cao B., Xie Y. (2020). Physiological-ecological responses of twelve herbaceous plant species under drought stress and evaluation of their drought resistance when planted in coal producting basis in arid windy and sandy areas. Acta Prataculturae Sinica.

[33] Wang J., Zhang X., Han Z., Feng H., Wang Y., Kang J., Han X., Wang L., Wang C., Li H. (2022). Analysis of physiological indicators associated with drought tolerance in wheat under drought and re-watering conditions. Antioxidants.

[34] Zhao L., Jin H.D., Cao X.Y., Deng W.H., Du L.J. (2023). Physiological response to drought stress and drought resistance of six *Helleborus orientlis* cultivars. Chin. J. Appl. Ecol..

[35] Zhou Y.X., Li P.T., Su J.S., Wang H.B., Fang W.M., Chen F.D., Zhang F. (2023). Evaluation of drought resistance in 37 accessions of chrysanthemum related species. J. Nanjing Agric. Univ..

[36] Ahmed S.F., Ahmed J.U., Hasan M., Mohi-Ud-Din M. (2023). Assessment of genetic variation among wheat genotypes for drought tolerance utilizing microsatellite markers and morpho-physiological characteristics. Heliyon.

[37] Bao X., Hou X., Duan W., Yin B., Ren J., Wang Y., Liu X., Gu L., Zhen W. (2023). Screening and evaluation of drought resistance traits of winter wheat in the North China Plain. Front. Plant Sci..

[38] Martínez I., Muñoz M., Acuña I., Uribe M. (2021). Evaluating the drought tolerance of seven potato varieties on volcanic ash soils in a medium-term trial. Front. Plant Sci..

[39] Shojaei S.H., Mostafavi K., Omrani A., Illés Á., Bojtor C., Omrani S., Mousavi S.M.N., Nagy J. (2022). Comparison of maize genotypes using drought-tolerance indices and graphical analysis under normal and humidity stress conditions. Plants.

[40] Sun F., Chen Q., Chen Q., Jiang M., Gao W., Qu Y. (2021). Screening of key drought tolerance indices for cotton at the flowering and boll setting stage using the dimension reduction method. Front. Plant Sci..

[41] Tiwari P.N., Tiwari S., Sapre S., Tripathi N., Payasi D.K., Singh M., Thakur S., Sharma M., Tiwari S., Tripathi M.K. (2023). Prioritization of physio-biochemical selection indices and yield-attributing traits toward the acquisition of drought tolerance in chickpea (*Cicer arietinum* L.). Plants.

[42] Yang J., Han D.X., Wang Y.J., Li M.D., Xi H.J., Abulati A., Liang X.L. (2019). Drought Tolerance Identification and Evaluation of 26 Maize Inbred Lines under Natural Drought Condition in Xinjiang. Xinjiang Agric. Sci..

[43] Zhou Y.Q., Yang Y.Z., Zhou W.Q., Lian X.R., Zhang Y.J., Wang X.R., Kou S.R., He H.J., Liu Z.X., Wang X.J. (2020). Evaluation and selection of drought resistance inbred lines of maize under drought stress. Agric. Res. Arid Areas.

[44] Zhao X.Z., Xu J.Y., Yu L.L., Gu Y.Z., Liu W.X., Qiu L.J. (2020). Field drought tolerance evaluation and excellent germplasm identification of soybean germplasm. Soybean Sci..

[45] Mahmood T., Iqbal M.S., Li H., Nazir M.F., Khalid S., Sarfraz Z., Hu D., Baojun C., Geng X., Tajo S.M. (2022). Differential seedling growth and tolerance indices reflect drought tolerance in cotton. BMC Plant Biol..

[46] Guo C., Zhu L., Sun H., Han Q., Wang S., Zhu J., Zhang Y., Zhang K., Bai Z., Li A. (2024). Evaluation of drought-tolerant varieties based on root system architecture in cotton (*Gossypium hirsutum* L.). BMC Plant Biol..

[47] Xiong S., Wang Y., Chen Y., Gao M., Zhao Y., Wu L. (2022). Effects of drought stress and rehydration on physiological and biochemical properties of four oak species in China. Plants.

[48] Islam M.J., Kim J.W., Begum M.K., Sohel M.A.T., Lim Y.S. (2020). Physiological and biochemical changes in sugar beet seedlings to confer stress adaptability under drought condition. Plants.

[49] Wang X.D. (2024). Evaluation of growth, physiological response, and drought resistance of different flue-cured tobacco varieties under drought stress. Front. Plant Sci..

[50] Zhang J., Huang D., Zhao X., Zhang M. (2021). Evaluation of drought resistance and transcriptome analysis for the identification of drought-responsive genes in *Iris germanica*. Sci. Rep..

[51] Li J., Abbas K., Wang L., Gong B., Hou S., Wang W., Dai B., Xia H., Wu X., Lü G. (2023). Drought resistance index screening and evaluation of lettuce under water deficit conditions on the basis of morphological and physiological differences. Front. Plant Sci..

